# Radiation-associated circulatory disease mortality in a pooled analysis of 77,275 patients from the Massachusetts and Canadian tuberculosis fluoroscopy cohorts

**DOI:** 10.1038/srep44147

**Published:** 2017-03-13

**Authors:** Van Tran, Lydia B. Zablotska, Alina V. Brenner, Mark P. Little

**Affiliations:** 1 Radiation Epidemiology Branch, National Cancer Institute, Bethesda, MD 20892-9778, USA; 2 Department of Epidemiology and Biostatistics, School of Medicine, University of California San Francisco, San Francisco, CA, USA; 3 Radiation Epidemiology Branch, National Cancer Institute, Bethesda, MD 20892-9778, USA

## Abstract

High-dose ionising radiation is associated with circulatory disease. Risks associated with lower-dose (<0.5 Gy) exposures remain unclear, with little information on risk modification by age at exposure, years since exposure or dose-rate. Tuberculosis patients in Canada and Massachusetts received multiple diagnostic x-ray fluoroscopic exposures, over a wide range of ages, many at doses <0.5 Gy. We evaluated risks of circulatory-disease mortality associated with <0.5 Gy radiation exposure in a pooled cohort of 63,707 patients in Canada and 13,568 patients in Massachusetts. Under 0.5 Gy there are increasing trends for all circulatory disease (*n* = 10,209; excess relative risk/Gy = 0.246; 95% CI 0.036, 0.469; *p* = 0.021) and for ischaemic heart disease (*n* = 6410; excess relative risk/Gy = 0.267; 95% CI 0.003, 0.552; *p* = 0.048). All circulatory-disease and ischaemic-heart-disease risk reduces with increasing time since exposure (*p* < 0.005). Over the entire dose range, there are negative mortality dose trends for all circulatory disease (*p* = 0.014) and ischaemic heart disease (*p* = 0.003), possibly due to competing causes of death over this dose interval.These results confirm and extend earlier findings and strengthen the evidence for circulatory-disease mortality radiation risk at doses <0.5 Gy. The limited information on well-known lifestyle/medical risk factors for circulatory disease implies that confounding of the dose trend cannot be entirely excluded.

The well-documented effects of ionising-radiation exposure include cancer^1^,^2^, and at higher doses, various types of tissue-reaction effect, in particular circulatory disease^3^. Circulatory diseases have been shown to be associated with radiation treatment of malignant^4^,^5^,^6^,^7^,^8^,^9^,^10^,^11^,^12^ and benign disease^13^. There is a substantial body of radiobiological data which suggests that certain inflammatory cytokines and adhesion markers thought to be involved in circulatory disease may be differentially up and down regulated at doses above and below ~0.5 Gy^14^, suggesting that attention should be restricted to the moderate dose range <0.5 Gy. However, risks associated with lower-dose (<0.5 Gy) exposures remain unclear. A recent report suggested that low dose-rate space radiation exposure may increase risk of circulatory disease^15^, although this finding is controverisal^16^. A meta-analysis of groups with mean exposure <0.5 Gy indicates excess circulatory-disease risk overall for two out of four disease endpoints, but suggests that inter-cohort heterogeneity for certain endpoints limits the causal interpretability of these findings^17^.

Previous analyses of long-term health effects with respect to circulatory disease mortality after exposure from x-ray fluoroscopy used in the course of treatment for tuberculosis have included cohorts from Canada^18^ and Massachusetts^19^. There is excess mortality risk for ischaemic heart disease (IHD) in the Canadian cohort after adjusting for dose fractionation^18^. There is decreasing excess mortality risk with increasing age at exposure and time since exposure, but an unexpected inverse dose fractionation effect^18^. Although there is little evidence of excess risk overall in the Massachusetts study, at doses <0.5 Gy there is evidence of excess mortality risk for all circulatory disease (*p* = 0.074) and IHD (*p* = 0.068)^19^; there are no indications of modifying effects of age at exposure, time since exposure or dose fractionation^19^.

The purpose of this paper is to investigate circulatory disease risk in the Canadian and Massachusetts tuberculosis fluoroscopy cohorts by using a pooled data set, with a focus on the effects of radiation at doses <0.5 Gy, and exploring also adjustments for age at exposure, time since exposure and radiation dose rate. Combining data will boost statistical power for certain rare outcomes such as hypertensive heart disease. Interpretation of the slightly different findings in the two cohorts will benefit from a unified methodological treatment.

## Results

In the pooled group exposed to <0.5 Gy, there are 58,676 persons, 48,068 from the Canadian cohort and 10,608 from the Massachusetts dataset (Table 1) and the mean cumulative lung dose is 0.18 Gy (range = 0.01, 0.50) (Supplementary Table S1). 17.4% (10,209/58,676) of patients died from circulatory disease (Table 2).

**Figure 1:**
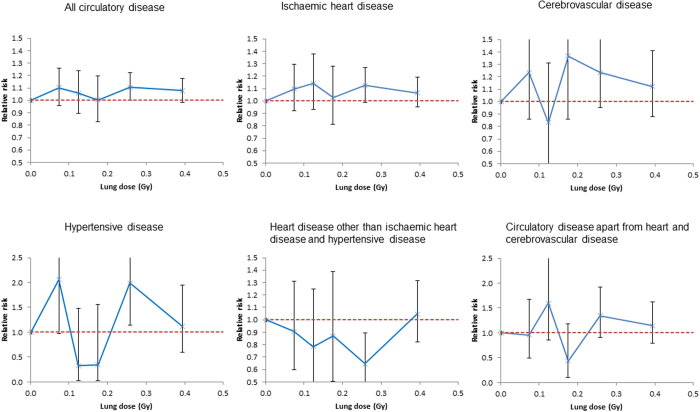
Relative risk estimates (and their 95% confidence intervals) against cumulative lagged dose (lagged by 5 years) for the restricted dose range [0, 0.5] Gy. We show results for the dose categories 0–0.049, 0.050–0.099, 0.100–0.149, 0.150–0.199, 0.200–0.299, 0.300–0.499 Gy. The dashed red line corresponds to relative risk =1.

**Figure 2:**
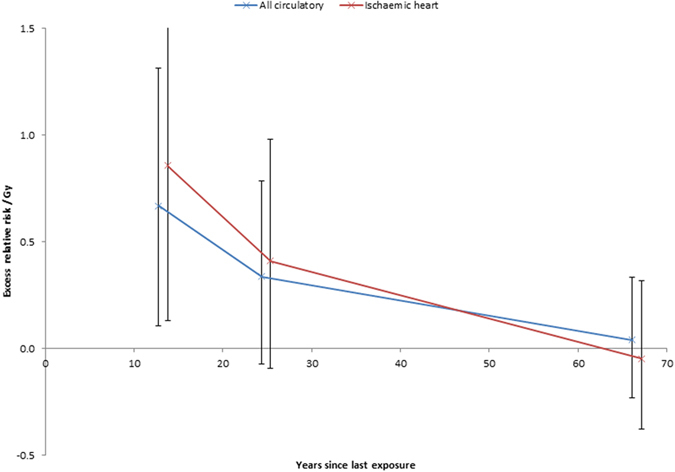
Variation of excess relative risk (+95% CI) with years since last exposure for all circulatory disease and ischaemic heart disease. We show results for the categories 0–19, 20–29 and ≥30 years since last exposure.

Under 0.5 Gy circulatory-disease mortality increases with dose (excess relative risk/Gy = 0.246; 95% CI 0.036, 0.469; *p* = 0.021) as also does IHD (excess relative risk/Gy = 0.267; 95% CI 0.003, 0.552; *p* = 0.048) (Table 3, Fig. 1). The Kaplan-Meier plots of Supplementary Fig. S1 demonstrate that the survival probabilities are very similar in the various dose groups until the age of 60 years, after which point they increasingly diverge. For no disease endpoint is there any significant modifying effect of age at first exposure, or radiation dose rate (*p* > 0.2). However, there is a pronounced (*p* < 0.005) reduction of relative risk for all circulatory disease and IHD with increasing time since last exposure (Table 4, Fig. 2); there are similar findings over the full dose range (Supplementary Table S3). There is no modifying effect of cohort on excess relative risk for any endpoint (*p* > 0.1) (results not shown).

Sensitivity analysis using 10-year (rather than 5-year) lag shows that there are positive dose trends for all circulatory disease (*p* = 0.018) and hypertensive heart disease (*p* = 0.027), as also to a lesser extent for IHD (*p* = 0.077) (Supplementary Table S4). Over the full dose range, the sensitivity analysis in Supplementary Table S4 and Supplementary Fig. S2 demonstrate that there is a decreasing trend in mortality risk with dose for all circulatory disease (*p* = 0.014) and IHD (*p* = 0.003). Removing the upper age limit (of 100 years) makes no difference to any results (results not shown). There is limited information on antibiotic use (Isoniazid, Streptomycin, Poly-aminosalicylic acid), and diabetes, all available only for a small part (1394/13,568) of the Massachusetts dataset (Table 1, Supplementary Table S2); information on alcohol consumption is available for the full Massachusetts cohort. These variables do not confound or otherwise modify the radiation dose response, but rather act as independent risk factors. When these variables are included in the model they have (as a group) highly significant independent effect, apart from radiation exposure, on all circulatory disease, IHD and heart disease apart from IHD and hypertensive disease (*p* < 0.01, results not shown); the adjusted trends with dose are very similar to those of the main analysis (Table 3, Supplementary Table S4). The significance of the ensemble of indicators is largely driven by the effects of alcohol consumption, diabetes and the indicator of the informative (for antibiotics and diabetes) subcohort of the Massachusetts dataset, which at least for all circulatory disease and IHD are conventionally statistically significant (*p* < 0.05) (Supplementary Table S4).

To assess the possibility of competing risks from causes of death other than circulatory disease, analyses of all circulatory-disease mortality using the subdistribution hazard (see Statistical Methods) yielded a risk estimate for dose <0.5 Gy that is consistent with the main analysis (excess relative risk/Gy = 0.339; *p* = 0.002 *vs* main analysis excess relative risk/Gy = 0.246; *p* = 0.021) (Supplementary Table S5). However, there is more discrepancy in the subdistribution hazard estimate for the full dose range (excess relative risk/Gy = −0.001; *p* = 0.933 *vs* main analysis excess relative risk/Gy = −0.024; *p* = 0.014) (Supplementary Table S5).

## Discussion

We found increased radiation dose-related excess mortality risk for all circulatory disease, IHD, and hypertensive heart disease in a pooled analysis of 58,676 tuberculosis patients from Canada and Massachusetts exposed to repeated x-ray fluoroscopies and with cumulative dose <0.5 Gy. This contrasts with analysis over the full dose range, when a negative trend in excess mortality risk with dose was observed for all circulatory disease, IHD, all deaths, and all deaths excluding circulatory disease. There is a strong reduction in radiation risk with increasing time since last exposure, but age at first exposure and radiation dose rate do not modify risk. Our findings <0.5 Gy are robust to a variant formulation using the subdistribution hazard, suggesting that competing risks from other causes of death are operating independently from circulatory disease. However, this is not the case over the full dose range.

Our results are similar to, but somewhat stronger than those of the previous analysis of the Massachusetts cohort^19^, which found no dose trends for any circulatory disease endpoint over the full dose range, and indications of increasing trends with dose for all circulatory disease and IHD <0.5 Gy (Supplementary Table S6). The previous analysis of the Canadian cohort^18^ reported weaker indications of excess IHD mortality risk <0.5 Gy (Supplementary Table S6). The Canadian study also reported an increasing trend for IHD mortality with dose in the entire dose range with an inverse risk modification by dose fractionation using 10-year lag, the evidence for which became much weaker when dose was restricted to less than 0.5 Gy, or when 5-year lag was used^18^. We did not have individual annual doses for the Massachusetts cohort, but analyses using average dose-rates for both cohorts did not find any modification by dose-rate for dose <0.5 Gy, or over the full dose range (Table 4); it may be that this somewhat different definition of radiation dose rate may account for the discrepancies in the dose-fractionation-modification findings from the previous analysis of the Canadian data^18^.

Our findings in relation to time-since-exposure modifications to relative risk (Table 4, Fig. 2, Supplementary Table S3) are similar to those in the Canadian TB cohort^18^ and in the Massachusetts data^19^ over the full dose range. The absence of any strong modification of risk by age at exposure (Table 4, Supplementary Table S3) contrasts with the pronounced inverse modification, with excess relative risk decreasing with increasing age at exposure in the Canadian TB cohort^18^ and the modification in the opposite direction, in the Massachusetts data over the full dose range^19^, the combination of which doubtless explains our null finding overall. In the Japanese atomic bomb survivor Life Span Study (LSS) cohort modifications to excess relative risk/Gy for age at exposure are consistent with those observed here for all endpoints^17^; the magnitude of the time since exposure adjustment in the LSS is inconsistent with the modification observed here for IHD (Table 4, Fig. 2), although consistent with the adjustment for other endpoints. The modification to excess relative risk/Gy with time after exposure for IHD and cerebrovascular disease (CeVD) in a US cohort of persons who received X-radiation treatment for peptic ulcer^13^ are consistent with those observed here. It may be significant that the type of radiation used in this study, moderate energy X-rays, is quite similar to the type of low energy, and largely unfiltered, fluoroscopy X-rays used here^20^, albeit for therapeutic rather than diagnostic purposes, and contrasts with the rather higher energy radiation to which nuclear workers and the LSS^21^,^22^ were exposed (Supplementary Table S6); it is well known that higher-energy gamma rays are less biologically effective per unit dose than X-rays in relation to a number of experimental endpoints, in particular chromosome translocations, dicentrics, cell transformation, cell killing, specific locus mutations and various others^23^. Also, a typical chest fluoroscopy in the period 1930–1950, when most of the dose in the cohort was incurred, would last about 15 s and patients would receive 0.01–0.10 Gy, and thus should not be considered a low dose-rate exposure^24^; in this respect it is similar to the peptic ulcer study^13^ and to the LSS^21^,^22^, and contrasts with the generally low dose-rate exposure in most of the other moderate/low dose cohorts listed in Supplementary Table S6.

A previous meta-analysis of groups exposed at low to moderate doses (mean whole-body dose <0.5 Sv) observed excess risk for IHD and stroke, and somewhat weaker evidence of excess risk for all circulatory disease excluding heart disease and stroke^17^. In particular, there is excess mortality and morbidity risk in the LSS^21^,^22^ and in various groups of nuclear workers^25^,^26^,^27^, which are consistent with our risk estimates for all circulatory disease and IHD <0.5 Gy (Supplementary Table S6). The findings over the full dose range are somewhat inconsistent, but as above, there are indications of interference from other causes of death over this full dose range in our data. Recent reviews have proposed biological mechanisms for the effects of radiation on circulatory disease^14^,^28^,^29^. At high therapeutic doses >5 Gy, damage to endothelial cells and capillaries may explain the adverse effects on the circulatory system^29^. At lower doses, 0.5–5 Gy, pro-inflammatory effects have been observed experimentally *in vivo* and *in vitro*, contrasting with anti-inflammatory effects at doses <0.5 Gy^14^,^28^,^30^. These different biological processes corresponding to different dose ranges suggest that at low and moderate doses, in particular <0.5 Gy, should be analysed separately from moderate and high doses. On the other hand, risk estimates in studies of medically exposed groups, which typically have organ doses much greater than 0.5 Gy^4^,^11^,^12^,^13^,^31^, are comparable to groups exposed at lower doses (Supplementary Table S6), suggesting that biological mechanism operating at high doses and dose rates may be similar to low and moderate doses and dose rates.

The present pooled analysis is the first such pooled analysis for any disease endpoints from the Canadian and Massachusetts tuberculosis fluoroscopy groups. Major strengths of the analysis are that it includes a large cohort that contains both sexes and various ages at exposure, and that has been followed through most of the 20^th^ century. Lung dose is evaluated, which should be a reasonable surrogate of dose to the heart^19^. The outcome and exposure information are both register-based, so most biases (e.g., due to misclassification of exposure or outcome) are unlikely. Although the combined dataset has information on smoking status and tuberculosis disease severity, both of which can modify circulatory disease risk, it lacks information on many other lifestyle factors, socio-economic status, medical risk factors for circulatory disease such as diabetes, obesity, and hypertension, also treatment-related factors for circulatory disease. Pooling data has resulted in the exclusion of variables such as alcohol consumption that is available in only one cohort^19^. There is limited information on alcohol consumption, antibiotic use (Isoniazid, Streptomycin, Poly-aminosalicylic acid), and diabetes, in general available only for a small part (*n* = 1394) of the Massachusetts dataset (Table 1, Supplementary Table S2), and all derived by questionnaire to the study subjects. Analysis adjusting for these variables suggested that for certain endpoints they are highly statistically significant; nevertheless the trends with dose were very similar to those of the main analysis (Table 3, Supplementary Table S4), implying that they do not confound the dose response. Although there is information on Isoniazid in the Canadian dataset^32^ the data is unfortunately unavailable for the present analysis. The significance of the effect of alcohol consumption is unsurprising in view of the similar findings in the previous analysis of the Massachusetts data^19^. The excess risk associated with diabetes is also unsurprising, as this has been consistently identified as a risk factor for circulatory disease^33^,^34^. However, in radiation-exposed groups that have such lifestyle or medical information, there is no evidence that lifestyle factors interact with radiation risk of circulatory disease^4^,^21^,^22^,^26^,^27^. It is not expected that, conditional on calendar period, treatment for circulatory disease would be associated with fluoroscopy dose, so that it is improbable that such factors would confound the dose response.

The previous meta-analysis suggested that if the association between low-level exposure to radiation and the risk of circulatory disease reflects an underlying causal relationship, linear in dose, then the overall excess risk of mortality after exposure to low doses or low dose-rates of radiation may therefore be about twice that currently assumed^17^. Since the excess relative risks that are derived here are consistent with those estimated previously, the implications for low dose radiation risk are unaltered.

In conclusion, our analysis of the combined Canadian and Massachusetts tuberculosis fluoroscopy cohorts corroborate certain key findings of previous analyses of the separate cohorts. For doses under 0.5 Gy, there are increasing trends with dose for IHD, hypertensive heart disease, and all circulatory disease. Although there is no positive dose trend for circulatory disease mortality risk in the full dose range, there are indications of interference from other causes of death over this range. Fluoroscopy is still a widely used method of diagnostic imaging^35^, in particular for interventional procedures, where doses can be considerable^36^, so these findings have considerable significance for the long term risks that may be associated with currently used methods of radiological diagnosis.

## Materials and Methods

### Cohort characteristics and follow-up

Medical records of patients treated for tuberculosis in all 46 Canadian institutions from 1930 to 1952 and in 12 Massachusetts hospitals from 1915 to 1968 were combined for this analysis. In the Canadian cohort multiple admissions to different institutions were identified by computerised record linkage of the patient records^37^, resulting in a cohort of 92,707 patients. Deaths in the cohort were ascertained via computerised record linkage with the Canadian Mortality Database. Because information on cause of death is available only since 1950, we included in the cohort only those n = 68,608 known to be alive at the beginning of 1950. Exclusions were made for those with incorrect age (n = 1653), invalid last contact status or year (n = 850), age of more than 100 years at the end of follow-up (n = 2392), and other record irregularities (n = 6), leaving a cohort of 63,707 patients for analysis in the Canadian cohort. Deaths in the Massachusetts cohort were retrospectively ascertained from the Vital Statistics Offices in the state of last known residence by linking to the mortality files of the Social Security Administration and the National Death Index and by contacting relatives and friends^38^. Vital status was also confirmed through records from the post office, motor vehicle departments, credit bureaus, and other sources^39^. Of the 13,716 members of the full Massachusetts cohort, exclusions were made for lack of adequate follow-up information (n = 144), and missing last exposure date (n = 4), leaving an analysis dataset of 13,568 persons. The combined cohort therefore contains 77,275 patients, 63,707 (82%) from the Canadian data and 13,568 (18%) from the Massachusetts study. More details about the methods used to assemble the separate cohorts can be found in earlier publications^39^,^40^,^41^. Details of individual dates of entry and exit from treatment, smoking status (unknown smoking status/ever smoker/never smoker), and most advanced stage of TB recorded (unknown/minimal/moderate/advanced) were abstracted from medical treatment records, and for some lifestyle data available for both cohorts (e.g., smoking) via interviews and questionnaires. For a group of 1502 members of the Massachusetts cohort (1472 with lung dose <0.5 Gy) with more than a single fluoroscopy the start and end of exposure dates are only known to be within a given calendar year; for these individuals the initial and final exposures were assumed to be separated by 4 months (~third of a year), the theoretically-expected separation for dates constrained to lie within a year. 404/13,568 cohort members with vital status not known after a certain date had follow-up censored then.

The study entry date is defined as the later of the entry date into the sanatoria for treatment beginning in 1915, and in the Canadian cohort, January 1, 1950. Follow-up ended on the earlier of the loss to follow-up, date of death, or December 31, 1987 for the Canadian cohort, or December 31, 2002 for the Massachusetts cohort.

The causes of death were recoded to the International Classification of Diseases, Ninth Revision (ICD-9)^42^. Our study focuses on deaths from all circulatory diseases and individual analysis of IHD, CeVD, hypertensive heart disease, heart disease apart from IHD and hypertensive heart disease, and other circulatory diseases, with associated ICD-9 codes given in Table 2. All information is for underlying cause of death. There is no information available to the investigators on contributing causes of death in either cohort.

### Dosimetry

Dosimetry methods for each cohort are detailed elsewhere^18^,^19^,^43^. In both cohorts, dose estimation accounted for the number of fluoroscopic screenings, data of typical fluoroscopic procedures during the period of exposure, and phantom studies^43^. Radiation doses to the lungs during fluoroscopic screenings were treated as surrogate doses to the heart and circulatory system. The fluoroscopy fields would encompass the heart more completely (i.e., the heart generally would be in the direct beam and also receive additional scattered radiation from the rest of the field); the fluoroscopy fields would not always encompass both lungs, so sometimes the lungs would be partially irradiated. Lung dose would therefore generally be expected to be slightly lower than heart dose, possibly by as much as a factor of 2^19^,^44^.

### Statistical methods

Person-years at risk were calculated for each stratum defined by cohort (Canada or Massachusetts), sub-cohort (Nova Scotia/non-Nova Scotia Canadian, Massachusetts I/II/III^39^), gender, tuberculosis stage, smoking status, attained age, calendar year at risk, cumulative lagged dose and dose rate categories; missing data were separately coded and incorporated into the person-year table. Sensitivity analyses were also conducted in which the upper age limit (of 100 years) was removed. In contrast to previous analyses of the Canadian data which used individual annual doses and actual duration of fluoroscopic procedures^18^, the current analyses assumed that dose was uniformly distributed over the exposure duration (=date discharge − date entry to sanatorium), so as to be comparable with the previous analysis of the Massachusetts cohort^19^ for which annual doses are not available. Dose rate was defined as the ratio: cumulative dose/exposure duration. For most analyses, the dose lag (and time from start of follow-up to entry into the analysis dataset) was 5 years, but a 10 year dose lag (and entry lag) was also assessed.

We modelled the relative risk (RR) for circulatory disease mortality using Poisson regression^45^, so that the expected number of deaths in stratum *i* (defined by the above non-dose variables) and dose group *j* with mean dose *D*_*ij*_ and mean person year *PY*_*ij*_ of follow-up is:


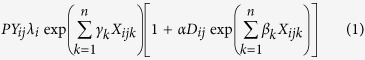


for some auxiliary modifying variables 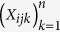, which included age at first exposure, time since last exposure and doserate. The model is linear in radiation dose, analogous to models previously used to assess circulatory disease in radiation exposed populations^13^,^17^,^22^. The excess relative risk per Gy *α*, the baseline mortality-rate modifying parameters *γ*_*k*_, the excess relative risk modifying parameters 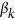, and the semi-parametric background rate *λ*_*i*_ were estimated from the model fit. In analysis adjusting for antibiotic use (Isoniazid, Streptomycin, Poly-aminosalicylic acid), and diabetes, we employ an indicator of the informative part of the Massachusetts cohort, and indicators for the presence of each of these exposures or medical conditions, adjusting the background risk via the parameters *γ*_*k*_; the results of the analysis adjusting for these variables and alcohol consumption (again adjusting the background risk via the parameters *γ*_*k*_) are provided in Supplementary Table S4. Sensitivity to the effects of competing risks from other types of mortality was assessed by fitting a Poisson model analogous to the subdistribution hazard of Fine and Gray^46^, which assumes that patients that died from causes other than circulatory disease were censored at the last day of follow-up in each cohort. Parameter estimation was by likelihood maximisation^45^ and was conducted in EPICURE^47^. All hypothesis tests were 2-sided. When possible, confidence intervals were estimated from the profile likelihood^45^, otherwise by Wald test inversion. Supplementary Table S6 cites results from a number of studies, some not already referenced in the main text^48^,^49^,^50^,^51^,^52^,^53^,^54^,^55^,^56^,^57^,^58^,^59^,^60^,^61^,^62^.

### Ethics approval

Ethics approval was previously obtained for the individual data sets, and extensions to this approval are not required for this study.

## Additional Information

**How to cite this article:** Tran, V. *et al*. Radiation-associated circulatory disease mortality in a pooled analysis of 77,275 patients from the Massachusetts and Canadian tuberculosis fluoroscopy cohorts. *Sci. Rep.* **7**, 44147; doi: 10.1038/srep44147 (2017).

**Publisher's note:** Springer Nature remains neutral with regard to jurisdictional claims in published maps and institutional affiliations.

## Supplementary Material

Supplementary Tables and Figures

## Figures and Tables

**Table 1:** Counts of patients for the Canadian and Massachusetts by demographic and exposure variables.

Descriptive characteristics	Categories	Numbers with lung dose <0.5 Gy/dose unrestricted		
		Canada	Massachusetts	Total
Canadian province	Nova Scotia	3431/4408	0/0	3431/4408
	non-Nova Scotia	44,637/59,299	0/0	44,637/59,299
Massachusetts subcohort	Massachusetts I	0/0	1101/1744	1101/1744
	Massachusetts II	0/0	5327/6986	5327/6986
	Massachusetts III	0/0	4180/4838	4180/4838
Gender	female	23,295/31,787	4934/6633	28,229/38,420
	male	24,773/31,920	5674/6935	30,447/38,855
Smoking status	never	2447/3456	2530/3390	4977/6846
	ever	7099/10,172	5108/6474	12,207/16,646
	unknown	38,522/50,079	2970/3704	41,492/53,783
Alcohol status	never	0/0	5392/7013	5392/7013
	ever	0/0	2633/3281	2633/3281
	unknown	0/0	2583/3274	2583/3274
Tuberculosis status	minimal	12,899/15,264	2187/2643	15,086/17,907
	moderate	15,299/22,696	3848/5229	19,147/27,925
	advanced	10,609/16,253	3832/4932	14,441/21,185
	unrecorded	9261/9494	741/764	10,002/10,258
Age at entry, year	0–19	10,447/14,249	1344/1804	11,791/16,053
	20–39	26,806/37,655	5220/7399	32,026/45,054
	40–59	8803/9765	3019/3322	11,822/13,087
	≥60	2012/2038	1025/1043	3037/3081
Age at first exposure, year	not screened	38,775/38,775	7229/7229	46,004/46,004
	0–19	1295/4500	412/1098	1707/5598
	20–39	6570/17,888	2207/4210	8777/22,098
	40–59	1363/2448	712/971	2075/3419
	≥60	65/96	49/60	113/156
Age at study exit, years	0–54	9222/11,696	2834/3562	12,056/15,258
	55–64	14,025/19,117	1635/2014	15,660/21,131
	65–74	14,470/20,818	2635/3286	17,105/24,104
	≥75	10,351/12,676	3504/4706	13,855/17,382
Cumulative lung dose, Gy	0	38,775/38,775	7754/7754	46,529/46,529
	>0–0.49	9293/9293	2854/2854	12,147/12,147
	0.50–0.99	0/5038	0/1123	0/6161
	1.00–1.99	0/6343	0/1241	0/7584
	≥2.00	0/4258	0/596	0/4854
Lung dose rate, Gy/year	0	38,775/38,775	7754/7754	46,529/46,529
	>0.0–0.19	2308/3141	1271/1562	3579/4703
	0.20–0.49	2585/9429	1040/2777	3625/12,206
	0.50–4.99	4387/12,349	543/1475	4930/13,824
	≥5.00	13/13	0/0	13/13
**Total**		**48,068**/**63,707**	**10,608**/**13,568**	**58,676**/**77,275**

In each cell we provide numbers of persons with cumulative dose <0.5 Gy (to the left of the oblique dash) and without restriction on dose (to the right of the oblique dash) in the respective cohorts (Canada, Massachusetts, total (Canada + Massachusetts)).

**Table 2:** Mortality counts by disease endpoint in the Canadian and Massachusetts cohorts, in relation to dose range, attained age range, lag period from start of follow-up to entry into analysis cohort.

Endpoint/type of circulatory disease	ICD9 codes	Number of deaths/person years		
		Canada	Massachusetts	Total
Lung dose < 0.5 Gy, age < 100, lag 5 years				
Cerebrovascular disease	430–438	1192	369	1561
Ischaemic heart disease	410–414	4876	1534	6410
Hypertensive heart disease	401–405	181	63	244
Heart disease apart from IHD + hypertensive	390–400, 406–409, 415–429	926	383	1309
All other circulatory disease apart from heart + cerebrovascular	439–459	518	167	685
All circulatory disease	390–459	7693	2516	10,209
Person years follow-up		1,179,270	247,711	1,426,981
Lung dose unrestricted, age < 100, lag 5 years				
Cerebrovascular disease	430–438	1481	472	1953
Ischaemic heart disease	410–414	6211	1947	8158
Hypertensive heart disease	401–405	234	89	323
Heart disease apart from IHD + hypertensive	390–400, 406–409, 415–429	1182	497	1679
All other circulatory disease apart from heart + cerebrovascular	439–459	659	211	870
All circulatory disease	390–459	9767	3216	12,983
Person years follow-up		1,599,120	345,921	1,945,041
Lung dose unrestricted, age unrestricted, lag 0 years				
Cerebrovascular disease	430–438	1585	493	2078
Ischaemic heart disease	410–414	6516	2086	8602
Hypertensive heart disease	401–405	286	102	388
Heart disease apart from IHD + hypertensive	390–400, 406–409, 415–429	1330	543	1873
All other circulatory disease apart from heart + cerebrovascular	439–459	701	222	923
All circulatory disease	390–459	10,418	3446	13,864
Person years follow-up		1,904,580	405,193	2,309,773

**Table 3:** Excess relative risk estimates for circulatory disease mortality endpoints by dose range using 5 year dose lag and 5 years from study entry to start of follow-up.

Dose range, Gy	Excess relative risk/Gy (+95% confidence intervals (CI))					
	All circulatory disease	IHD	Cerebrovascular	Hypertensive	Heart disease apart from IHD and hypertensive	All other circulatory diseases apart from heart and cerebrovascular
0–0.10	1.135 (−0.494, 2.900)	1.371 (−0.708, 3.664)	1.998 (−2.102, 7.027)	15.340 (2.298, 35.100)	−1.586 (−4.990, 2.759)	−1.724 (−6.643, 5.339)
*p*-value^a^	0.177	0.204	0.367	0.015	0.443	0.589
0–0.20	0.322 (−0.444, 1.151)	0.618 (−0.353, 1.687)	0.979 (−1.043, 3.453)	−0.448 (−4.791, 7.003)	−1.121 (−2.726, 0.914)	−0.564 (−3.212, 3.143)
*p*-value^a^	0.421	0.221	0.370	0.882	0.257	0.733
0–0.30	0.371 (−0.006, 0.772)	0.490 (0.013, 1.006)	0.915 (−0.101, 2.109)	3.033 (0.169, 7.216)	−1.292 (−1.998, −0.417)	0.856 (−0.622, 2.749)
*p*-value^a^	0.054	0.044	0.080	0.035	0.006	0.283
0–0.40	0.268 (0.002, 0.551)	0.337 (0.001, 0.699)	0.661 (−0.059, 1.499)	1.065 (−0.746, 3.711)	−0.588 (−1.131, 0.064)	0.540 (−0.531, 1.892)
*p*-value^a^	0.049	0.049	0.074	0.290	0.075	0.352
0–0.50	0.246 (0.036, 0.469)	0.268 (0.003, 0.552)	0.441 (−0.119, 1.090)	1.121 (−0.351, 3.228)	−0.226 (−0.679, 0.307)	0.507 (−0.322, 1.541)
*p*-value^a^	0.021	0.047	0.129	0.155	0.385	0.253

^a^*p*-value for departure of trend from null. All models adjust for cohort/sub-cohort, gender, smoking status, tuberculosis status, attained age, calendar year at risk by stratification. All CI are profile-likelihood based.

**Table 4:** Excess relative risk (ERR) estimates (and 95% confidence intervals (CI)) and adjustment factors for circulatory disease mortality endpoints (and 95% CI) for 0–0.5 Gy dose range in models that adjust for (a) age at first exposure, (b) years since last exposure or (c) radiation dose rate.

Type of adjustment to ERR/Gy	Excess relative risk/Gy (+95% CI)/% adjustment (+95% CI)/*p*-value					
	All circulatory disease	IHD	Cerebrovascular	Hypertensive	Heart disease apart from IHD and hypertensive	All other circulatory diseases apart from heart and cerebrovascular
Linear ERR/Gy adjusted for age at first exposure	0.165 (0.013, 0.478)	0.214 (−0.088^a^, 0.544)	0.510 (−0.159^a^, 1.242)	1.166 (−0.688^a^, 3.456)	−0.236 (−0.664, 0.309^a^)	0.461 (−0.621^a^, 1.688)
Age at first exposure adjustment (% change in ERR/Gy per year of age at first exposure)	−13.0 (−25.8, 8.5)	3.2 (−32.5, 25.9)	−4.3 (−18.3^a^, 12.1^a^)	−0.8 (−14.2^a^, 14.6^a^)	3.6 (−7.9^a^, 16.7^a^)	1.2 (−13.2^a^, 17.9^a^)
*p*-value^b^ for modification of ERR	0.251	0.552	0.593	0.890	0.353	0.899
Linear ERR/Gy adjusted for time since last exposure	0.272 (−0.024^a^, 0.582)	0.215 (−0.109^a^, 0.659)	0.540 (−0.203^a^, 1.338)	1.047 (−0.824^a^, 3.179)	−0.215 (−0.701, 0.408)	0.007 (−0.059^a^, 1.387)
Years since last exposure adjustment (% change in ERR/Gy per year since last exposure)	−10.5 (−49.2, −3.9)	−14.7 (−42.6, −6.4)	−6.1 (−17.3^a^, 6.5^a^)	1.6 (−8.5^a^, 13.0^a^)	0.6 (−14.2^a^, 17.9^a^)	21.6 (−7.9^a^, 60.6^a^)
*p*-value^b^ for modification of ERR	0.002	< 0.001	0.402	0.693	0.899	0.244
Linear ERR/Gy adjusted for dose rate	0.247 (0.036, 0.470)	0.268 (−0.014^a^, 0.551)	0.467 (−0.101, 1.117)	1.108 (−0.633^a^, 3.220)	−0.228 (−0.678, 0.379^a^)	0.500 (−0.467^a^, 1.535)
Dose rate adjustment (% change in ERR/Gy per Gy/year)	3.11 (−95.85, 67.50)	−19.32 (−99.28, 60.32)	32.51 (−30.36^a^, > 100^a^)	−20.05 (−98.62^a^, > 100^a^)	−59.51 (−99.98^a^, > 100^a^)	−22.65 (−99.63^a^, > 100^a^)
*p*-value^b^ for modification of ERR	0.950	0.684	0.565	0.877	0.570	0.847

^a^Wald-based CI.

^b^2-sided *p*-value for departure of trend from null.

All models adjust for cohort/sub-cohort, gender, smoking status, tuberculosis status, attained age, calendar year at risk by stratification. All CI are profile-likelihood based. The adjustments for age at first exposure, years since last exposure and dose rate are centered at the person-year-weighted mean values for the <0.5 Gy data, namely 27.98 years, 25.09 years and 0.61 Gy/year, respectively. 5 year dose lag and period from entry to start of follow-up. Unless otherwise stated all CI are based on the profile likelihood.
