# Supplementary material for: Radiation-associated circulatory disease mortality in a pooled analysis of 77,275 patients from the Massachusetts and Canadian tuberculosis fluoroscopy cohorts
Source: Sci Rep. 2017 Mar 13;7:44147. doi: 10.1038/srep44147 (PMC5347030; doi:10.1038/srep44147)

Radiation-associated circulatory disease mortality in a pooled analysis of 77,275 patients from the Massachusetts and Canadian tuberculosis fluoroscopy cohorts

Van Tran, Lydia B Zablotska, Alina V Brenner, Mark P Little

SUPPLEMENTARY TABLES AND FIGURES

Supplementary Table S1. Summary statistics of patients who were irradiated (lung dose > 0) in the Canadian and Massachusetts cohorts (*n*=11,339 lung dose < 0.5 Gy, *n*=29,526 lung dose unconstrained). Analysis cohort is restricted to those that contribute non-zero person-years of follow-up, after 5 year lag.

Variable	Canada			Massachusetts			Total	
	Lung dose < 0.5 Gy							
	mean	median	range	mean	median	range	mean	range
Lung dose, Gy	0.18	0.16	(0.01, 0.50)	0.19	0.14	(0.01, 0.50)	0.18	(0.01, 0.50)
Dose rate, Gy/year	0.59	0.49	(0.00, 9.17)	0.68	0.25	(0.00, 4.33)	0.61	(0.00, 9.17)
Age at first exposure, year	29.44	27.31	(2.56, 80.64)	31.39	29.16	(9.98, 69.83)	29.82	(2.56, 80.64)
Minimum time since last exposure, years	3.33	4.25	(-18.92, 22.17)	5.13	5.44	(-35.03, 21.98)	3.69	(-35.03, 22.17)
Maximum time since last exposure, years	35.59	38.31	(-0.15, 57.78)	35.63	38.92	(-0.03, 69.65)	35.59	(-0.15, 69.65)
Number of fluoroscopy screenings	15.08	13.00	(0.99, 48.00)	18.28	13.00	(1.00, 76.00)	15.71	(0.99, 76.00)
Duration of exposure, years	0.67	0.54	(0.02, 20.91)	0.91	0.33	(0.00, 23.00)	0.72	(0.00, 23.00)
	Lung dose unconstrained							
	mean	median	range	mean	median	range	mean	range
Lung dose, Gy	1.16	0.81	(0.01, 27.77)	0.90	0.58	(0.01, 8.56)	1.12	(0.01, 27.77)
Dose rate, Gy/year	0.56	0.50	(0.00, 9.17)	0.54	0.34	(0.00, 4.33)	0.56	(0.00, 9.17)
Age at first exposure, year	27.45	25.47	(1.97, 80.64)	28.70	26.56	(4.93, 69.83)	27.66	(1.97, 80.64)
Minimum time since last exposure, years	1.59	2.54	(-32.25, 22.17)	3.56	4.64	(-35.03, 21.98)	1.92	(-35.03, 22.17)
Maximum time since last exposure, years	34.95	37.40	(-5.98, 57.78)	37.44	42.07	(-0.03, 69.65)	35.36	(-5.98, 69.65)
Number of fluoroscopy screenings	92.70	65.06	(0.99, 2041.05)	83.00	55.00	(1.00, 833.00)	91.09	(0.99, 2041.05)
Duration of exposure, years	2.60	1.71	(0.02, 35.38)	2.68	2.00	(0.00, 25.00)	2.61	(0.00, 35.38)

Supplementary Table S2. Numbers of persons taking antibiotics and with diabetes in informative subset (*n*=1394) of Massachusetts cohort.

	Lung dose < 0.5 Gy		Lung dose unrestricted	
Condition	number	percentage	number	percentage
Isoniazid	218	23.0	344	24.7
Streptomycin	400	42.2	542	38.9
Para-aminosalicylic acid	336	35.4	458	32.9
Diabetes	42	4.4	61	4.4
All informative individuals	948	100.0	1394	100.0

Supplementary Table S3. Excess relative risk (ERR) estimates (+95% confidence intervals (CI)) for circulatory disease mortality endpoints for the full dose range in models that adjust for (a) age at first exposure, (b) time since last exposure or (c) radiation dose rate. These adjustments are centered at the person-year weighted mean values for the full dose range data, namely 26.26 years, 23.88 years and 0.56 Gy/year. 5 year dose lag and period from entry to start of follow-up. Unless otherwise stated all CI are based on the profile likelihood.

	Excess relative risk / Gy (+95% CI)					
Sensitivity analysis	All circulatory disease	IHD	Cerebrovascular	Hypertensive	Heart disease apart from IHD and hypertensive	All other circulatory diseases apart from heart and cerebrovascular
Linear ERR adjusted for age at first exposure	-0.026 (-0.044, -0.007)	-0.037 (-0.062[a], -0.012[a])	-0.017 (-0.071[a], 0.043)	-0.036[b] (-0.160[a], 0.088[a])	-0.010 (-0.071[a], 0.043)	0.056 (-0.050[a], 0.172)
Age at first exposure adjustment (% change in ERR / Gy per year of age at first exposure)	-4.9 (-14.6[a], 3.3)	-5.1 (-13.4[a], 3.5)	-2.6 (-34.3[a], 44.5[a])	-4.6[b] (-41.5[a], 55.5[a])	-6.4 (-57.7[a], 107.3[a])	-0.4 (-16.3[a], 18.4[a])
p-value[c] for modification of ERR	0.198	0.216	0.735	0.599[b]	0.843	0.950
Linear ERR adjusted for time since last exposure	-0.015 (-0.033, 0.001[a])	-0.028 (-0.049[a], -0.007[a])	-0.007[b] (-0.038[a], 0.023[a])	-0.009[b] (-0.089[a], 0.070[a])	-0.001[b] (-0.014[a], 0.011[a])	0.051 (-0.057[a], 0.170)
Years since last exposure adjustment (% change in ERR / Gy per year since last exposure)	7.5 (3.3, 11.8[a])	6.2 (2.7, 9.0[a])	10.0[b] (-3.1[a], 24.9[a])	9.4[b] (-16.6[a], 43.6[a])	14.5[b] (-14.9[a], 53.9[a])	1.4 (-13.0[a], 18.3[a])
p-value[c] for modification of ERR	0.001	0.002	0.082[b]	0.444[b]	0.184[b]	0.879

Linear ERR / Gy adjusted for dose rate	-0.025 (-0.043[a], -0.007[a])	-0.037 (-0.056[a], -0.016)	-0.014[b] (-0.065[a], 0.036[a])	-0.034[b] (-0.148[a], 0.159)	-0.017 (-0.056, 0.043[a])	0.058 (-0.037[a], 0.163)
Dose rate adjustment (% change in ERR / Gy per Gy / year)	69.32 (-74.59, 83.95[a])	62.04 (<0[a], 72.65[a])	64.26[b] (-66.55[a], >100[a])	51.33[b] (-92.75[a], >100[a])	-95.69 (-100.00[a], >100[a])	97.41 (-25.67[a], >100[a])
p-value[b] for modification of ERR	0.179	0.078	0.525[b]	0.704[b]	0.370	0.418

[a]Wald-based CI.
[b]failure for the model to converge by maximum likelihood estimation
[c]2-sided *p*-value for departure of trend from null.
All models adjust for cohort, sub-cohort, gender, smoking status, tuberculosis status, attained age, calendar year at risk by stratification.

Supplementary Table S4. Excess relative risk / Gy (+95% confidence intervals) for circulatory disease mortality endpoints for various sensitivity analyses. Unless otherwise stated the dose lag (and period from entry to start of follow-up) is set to 5 years, cumulative lung dose < 0.5 Gy, and all confidence intervals are based on the profile likelihood.

Variables	All circulatory disease	IHD	Cerebrovascular	Hypertensive	Heart disease apart from IHD and hypertensive	All other circulatory diseases apart from heart and cerebrovascular
	10 year dose lag, follow-up 10 years after entry					
Excess relative risk	0.262 (0.044, 0.493)	0.246 (-0.025, 0.537)	0.391 (-0.179, 1.054)	2.043 (0.194, 4.730)	-0.107 (-0.600, 0.477)	0.515 (-0.328, 1.575)
p-value[a]	0.018	0.077	0.190	0.027	0.700	0.253
Deaths	9284	5872	1424	198	1158	632
	Lung dose unlimited					
Excess relative risk	-0.024 (-0.042, -0.005)	-0.037 (-0.060[b], -0.013)	-0.014 (-0.067[b], 0.044)	-0.035 (-0.152[b], 0.153)	-0.010 (-0.064[b], 0.043)	0.055 (-0.028, 0.164)
p-value[a]	0.014	0.003	0.605	0.665	0.689	0.219
Deaths	12,983	8158	1953	323	1679	870
	Additional adjustment for alcohol consumption, antibiotic use (as in Supplementary Table S2) and diabetes (information only available within (parts of) the Massachusetts data)					
Excess relative risk	0.253 (0.042, 0.476)	0.286 (0.020, 0.572)	0.435 (-0.125, 1.083)	1.147 (-0.333, 3.266)	-0.240 (-0.691, 0.291)	0.511 (-0.320, 1.547)
p-value[a, c]	0.018	0.035	0.134	0.147	0.355	0.250
p-value for alcohol[d]	<0.001	<0.001	0.201	0.242	0.014	0.261
p-value for indicator of information on antibiotics/diabetes[d]	<0.001	<0.001	0.012	0.078	0.001	0.020
p-value for diabetes[d]	0.027	0.017	0.407	0.569	0.377	0.248
p-value for antibiotics[d]	0.807	0.328	0.692	0.661	0.689	0.585
Deaths	10,209	6410	1561	244	1309	685

[a]2-sided *p*-value for departure of linear dose trend from null.
[b]Wald-based CI.
[c]2-sided *p*-value for improvement in fit from adding linear trend with dose to the stratified model adjusted for alcohol use, availability of information on antibiotic use and diabetes, diabetes, and antibiotic use (Isoniazid, Streptomycin, Para-aminosalicylic acid).
[d]2-sided *p*-value for improvement in fit from adding successively indicators for (a) alcohol use, (b) availability of information on antibiotic use and diabetes, (c) diabetes, (d) antibiotic use (Isoniazid, Streptomycin, Para-aminosalicylic acid) to the baseline stratified model.
All models adjust by default for cohort, sub-cohort, gender, smoking status, tuberculosis status, attained age, calendar year at risk by stratification. Models in the bottom-most part of the table also adjust (by varying the background model (1) parameters γ_k rather than via stratification) for alcohol consumption, antibiotic use (as in Supplementary Table S2) and diabetes, with adjustment also for membership of the informative part of the Massachusetts cohort.

Supplementary Table S5. Competing risk analysis for all circulatory disease mortality risk from the full dose range and < 0.5 Gy lung dose range. 5 year dose lag and period from entry to start of follow-up.

	All circulatory disease	All deaths	Deaths other than circulatory disease	All circulatory disease with all other deaths censored at date of last follow-up in each cohort
	Lung dose < 0.5 Gy			
Excess relative risk / Gy + 95% CI	0.246 (0.036, 0.469)	-0.023 (-0.146, 0.104)	-0.021 (-0.031, -0.009)	0.339 (0.123, 0.565)
p-value	0.021	0.716	<0.001	0.002
number of deaths	10209	25022	14813	10209
	Lung dose unrestricted			
Excess relative risk / Gy + 95% CI	-0.024 (-0.042, -0.005)	-0.185 (-0.334, -0.029)	-0.019 (-0.031, -0.005)	-0.001 (-0.021, 0.021)
p-value	0.014	0.021	0.010	0.929
number of deaths	12983	32581	19598	12983

All models adjust for cohort/sub-cohort, gender, smoking status, tuberculosis status, attained age, calendar year at risk by stratification.

Supplementary Table S6. Excess relative risk estimates for circulatory disease in the present study and various low and moderate dose studies (adapted from Little *et al*. [17,48]). All data are in relation to the underlying cause of death, unless otherwise indicated.

Data	Reference	Average heart/brain dose (range) (Sv)	Numbers in cohort (person years follow-up)	Endpoint (mortality unless otherwise indicated)	Excess relative risk Sv^{-1} (and 95% confidence intervals)
**Diagnostic Medical Studies**					
Present study		1.13 (0.01-27.77)[a]	77,274 (1,904,580)	IHD (ICD9 410–414) <0.5 Gy	0.268 (0.003, 0.552)
				Hypertensive heart disease (ICD9 401–405) <0.5 Gy	1.121 (-0.351, 3.228)
				CeVD (ICD9 430–438) <0.5 Gy	0.441 (-0.119, 1.090)
				All circulatory disease (ICD9 390–459) <0.5 Gy	0.246 (0. 036, 0.469)
				IHD (ICD9 410–414) full dose range	-0.037 (-0.060, -0.013)
				Hypertensive heart disease (ICD9 401–405) full dose range	-0.035 (-0.152, 0.153)
				CeVD (ICD9 430–438) full dose range	-0.014 (-0.067, 0.044)
				All circulatory disease (ICD9 390–459) full dose range	-0.024 (-0.042, -0.005)
Massachusetts tuberculosis fluoroscopy	Little *et al.* [19]	0.36 (0-8.56)[a,b]	13,572 (345,948)	IHD (ICD9 410–414) <0.5 Gy	0.465 (-0.032, 1.034)[a]
				Hypertensive heart disease (ICD9 401–405) <0.5 Gy	0.801 (-1.266, 4.638)[a]
				CeVD (ICD9 430–438) <0.5 Gy	0.343 (-0.536, 1.473)[b]
				All circulatory disease (ICD9 390–459) <0.5 Gy	0.345 (-0.032, 0.764)[a]
				IHD (ICD9 410–414) full dose range	-0.077 (-0.130, -0.012)[a]
				Hypertensive heart disease (ICD9 401–405) full dose range	0.357 (-0.043, 1.030)[a]
				CeVD (ICD9 430–438) full dose range	0.132 (-0.088, 0.415)[b]
				All circulatory disease (ICD9 390–459) full dose range	-0.023 (-0.067, 0.028)[a]
Canadian tuberculosis fluoroscopy	Zablotska *et al.*[18]	0.79 (0–11.60)[a]	63,707 (1,902,252)	IHD (ICD9 410–414, 429.2)	0.007 (-0.044, 0.072)[c]
				Hypertensive disease and other non-CeVD (ICD9 390–409, 415–429.1, 429.3–429.9, 439–459)	0.027 (-0.064, 0.167)[c]
				All circulatory disease apart from CeVD (ICD9 390–429, 439–459)	0.020 (-0.025, 0.074)[c]
**Therapeutic medical studies (non-malignant disease)**					
Peptic ulcer study	Little *et al.* [13]	1.01 (0.0 – 6.20)	3600 (76,571.7)	IHD ICD9 410-414 using heart dose	0.102 (0.039, 0.174)
				IHD ICD9 410-414 using thyroid dose	1.696 (0.651, 2.907)
				IHD ICD9 410-414 using kidney dose	0.033 (0.012, 0.056)

				IHD ICD9 410-414 using pancreas dose	0.020 (0.008, 0.035)
				CeVD ICD9 430-438 using heart dose	0.028 (-0.085, 0.186)
				CeVD ICD9 430-438 using thyroid dose	0.422 (-1.455, 3.039)
				CeVD ICD9 430-438 using brain dose	2.649 (-8.912, 18.740)
				All other circulatory disease ICD9 390-409, 415-429, 439-459 using heart dose	0.050 (-0.053, 0.194)
				All circulatory disease ICD9 390-459 using heart dose	0.082 (0.031, 0.140)
**Japanese atomic bomb survivors**					
Mortality	Shimizu *et al.* [22]	0.1 (0 - 4)[d]	86,611 (n.a.)	IHD (ICD9 410-414)	0.02 (-0.10, 0.15)
				Myocardial infarction (ICD9 410)	0.00 (-0.15, 0.18)
				Hypertensive heart disease (ICD9 402, 404)	0.37 (0.08, 0.72)
				Rheumatic heart disease (ICD9 393-398)	0.86 (0.25, 1.72)
				Heart failure (ICD9 428)	0.22 (0.07, 0.39)
				Other heart disease (ICD9 390-392, 415-427, 429)	-0.01 (-0.21, 0.24)
				Hypertensive disease without heart disease (ICD9 401, 403, 405)	0.07 (-0.22, 0.55)
				Heart disease total (ICD9 393-429 excluding 401, 403, 405)	0.18 (0.11, 0.25)[e]
				Cerebral infarction (ICD9 433,434)	0.04 (-0.10, 0.20)
				Cerebral hemorrhage (ICD9 431)	0.05 (-0.06, 0.17)
				Subarachnoid hemorrhage (ICD9 430)	0.30 (-0.04, 0.76)
				Other or unspecified cerebrovascular disease	0.16 (0.01, 0.34)
				CeVD total (ICD9 430-438)	0.12 (0.05, 0.19)[e]
				Circulatory disease apart from heart disease and stroke (ICD9 390-392, 401, 403, 405, 439-459)	0.58 (0.45, 0.72)[e]
				Other circulatory disease (ICD9 399-400, 406-409, 439-459)	-0.01 (<-0.01, 0.34)
				All circulatory disease (ICD9 390-459)	0.15 (0.10, 0.20)[e]
Morbidity	Yamada *et al.* [21]	0.1 (0 - 4)[e, f]	10,339 (n.a.)	Hypertension incidence, 1958-1998 (ICD9 401)	0.05 (-0.01, 0.10)
				Hypertensive heart disease incidence, 1958-1998 (ICD9 402, 404)	-0.01 (-0.09, 0.09)
				IHD incidence, 1958-1998 (ICD9 410-414)	0.05 (-0.05, 0.16)
				Myocardial infarction incidence, 1964-1998 (ICD9 410)	0.12 (-0.16, 0.60)
				Occlusion incidence, 1958-1998 (ICD9 433, 434)	0.06 (-0.11, 0.30)
				Aortic aneurysm incidence, 1958-1998 (ICD9 441, 442)	0.02 (-0.22, 0.41)
				Stroke incidence, 1958-1998 (ICD9 430, 431, 433, 434, 436)	0.07 (-0.08, 0.24)

Morbidity *in utero*	Tatsukawa *et al.* [49]	0.001 (0-1.79)	506 (9,265)	Hypertension<br>Non-fatal stroke or myocardial infarction	0.20 (-0.39, 1.38)<br>-0.91 (-1.00, 79.3)
Morbidity in childhood	Tatsukawa *et al.* [49]	0.13 (0-3.53)	1,053 (20,216)	Hypertension<br>Non-fatal stroke or myocardial infarction	0.15 (-0.01, 0.34)<br>0.72 (0.24, 1.40)
**Occupational studies**					
Mayak workers	Moseeva *et al.* [50]<br>Azizova *et al.* [51]	0.62 ± 0.80 (males)[g]<br>0.51 ± 0.68 (females)[d]	22,377 (447,281)	IHD morbidity (ICD9 410-414)	0.14 (0.08, 0.21)[h]
				IHD morbidity (ICD9 410-414)	0.14 (0.08, 0.21)[c]
				IHD morbidity (ICD9 410-414)	0.16 (0.10, 0.24)[i]
			22,377 (836,048)	IHD mortality (ICD9 410-414)	0.05 (-0.01, 0.13)[h]
				IHD mortality (ICD9 410-414)	0.05 (-0.01, 0.13)[c]
				IHD mortality (ICD9 410-414)	0.05 (-0.01, 0.13)[i]
			18,856 (341,663)	CeVD morbidity (ICD9 430-438)	0.497 (0.393, 0.601)[h]
				CeVD morbidity (ICD9 430-438)	0.529 (0.415, 0.642)[c]
				CeVD morbidity (ICD9 430-438)	0.572 (0.450, 0.695)[i]
			18,856 (272,525)	CeVD mortality (ICD9 430-438)	0.057 (-0.046, 0.161)[h]
				CeVD mortality (ICD9 430-438)	0.064 (-0.042, 0.170)[c]
				CeVD mortality (ICD9 430-438)	0.076 (-0.033, 0.186)[i]
Chernobyl emergency workers	Ivanov *et al.* [52]	0.109 (0 - >0.5)	61,017 (n.a.)	Hypertension (ICD10 I10-I15) morbidity	0.26 (-0.04, 0.56)
				Essential hypertension (ICD10 I10) morbidity	0.36 (0.005, 0.71)
				Hypertensive heart disease (ICD10 I11) morbidity	0.04 (-0.36, 0.44)
				IHD (ICD10 I20-I25) morbidity	0.41 (0.05, 0.78)
				Acute myocardial infarction (ICD10 I21) morbidity	0.19 (-0.99, 1.37)
				Other acute IHD (ICD10 I24) morbidity	0.82 (-0.62, 2.26)
				Angina pectoris (ICD10 I20) morbidity	0.26 (-0.19, 0.71)
				Chronic IHD (ICD10 I25) morbidity	0.20 (-0.23, 0.63)
				Other heart disease (ICD10 I30-I52) morbidity	-0.26 (-0.81, 0.28)
				CeVD (ICD10 I60-I69) morbidity	0.45 (0.11, 0.80)
				Morbidity from diseases of arteries, arterioles and capillaries (ICD10 I70-I79)	0.47 (-0.15, 1.09)
				Morbidity from diseases of veins, lymphatic vessels and lymph nodes (ICD10 I80-I89)	-0.26 (-0.70, 0.18)
				All circulatory disease (ICD10 I00-I99) morbidity	0.18 (-0.03, 0.39)

Chernobyl emergency workers	Kaschcheev *et al.* [53]	0.161 (0.0001 - 1.24)	53,772 (958,540.5)	CeVD (ICD10 I60-I69) morbidity after no diabetes	0.35 (0.18, 0.53)
				CeVD (ICD10 I60-I69) morbidity after diabetes	1.29 (0.63, 1.94)
				CeVD (ICD10 I60-I69) morbidity after no atherosclerosis	0.43 (0.25, 0.62)
				CeVD (ICD10 I60-I69) morbidity after atherosclerosis	0.50 (0.09, 0.90)
				CeVD (ICD10 I60-I69) morbidity after no hypertensive disease	0.38 (0.08, 0.68)
				CeVD (ICD10 I60-I69) morbidity after hypertensive disease	0.48 (0.27, 0.68)
				CeVD (ICD10 I60-I69) morbidity after no IHD	0.41 (0.14, 0.68)
				CeVD (ICD10 I60-I69) morbidity after IHD	0.47 (0.25, 0.69)
				CeVD (ICD10 I60-I69) morbidity after no concomitant disease	0.19 (-0.99, 1.37)
				CeVD (ICD10 I60-I69) morbidity	0.45 (0.28, 0.62)
German uranium miner study	Kreuzer *et al.* [54]	0.041 (0 – 0.909)[j]	58,982 (2,180,639)	All circulatory disease (ICD10 I00-I99)	-0.13 (-0.38, 0.12)
				IHD (ICD10 I20-I25)	-0.03 (-0.38, 0.32)
				CeVD (ICD10 I60-I69)	0.44 (-0.16, 1.04)
EdF workers	Laurent *et al.* [55]	0.0215 (0 – 0.6)	22,393 (440,984)	IHD	4.1 (-2.9, 13.7)[k]
				CeVD	17.4 (0.2, 43.9)[k]
				All circulatory disease	2.7 (-2.3, 9.1)[k]
Eldorado uranium miners and processing (male) workers	Lane *et al.* [56]	0.0522 (<0.0234 – >0.1215)	16,236 (508,673)	IHD	0.15 (-0.14, 0.58)
				Stroke	-0.29 (<-0.29, 0.27)
				All other circulatory disease	0.07 (<-0.33, 0.77)
BNFL workers	McGeoghegan *et al.* [25]	0.0569 (0 – >0.729)	38,779 (1,081,570)	IHD (ICD9 410-414)	0.70 (0.37, 1.07)[e, i, k]
				CeVD (ICD9 430-438)	0.66 (0.17, 1.27)[e, i, k]
				Other circulatory diseases (ICD9 390-398, 415-429, 440-459)	0.83 (-0.10, 1.12)[i, k]
				Circulatory diseases apart from CeVD (ICD9 390-429, 439-459)	0.72 (0.39, 1.10)[i, k]
				All circulatory disease (ICD9 390-459)	0.54 (0.30, 0.82)[e, i, k]
3rd Analysis of UK National Registry for Radiation Workers	Muirhead *et al.* [57]	0.0249 (<0.01 - >0.4)	174,541 (3.9 x 10^6)	All circulatory disease (ICD9 390-459)	0.251 (-0.01, 0.54)
				Circulatory disease not strongly related to smoking (ICD9 390-409, 415-440, 442-459)	0.280 (-0.19, 0.85)
				Aortic aneurysm (ICD9 441)	-0.132 (-1.29, 1.92)
				IHD (ICD9 410-414)	0.259 (-0.05, 0.61)
				CeVD (ICD9 430-438)	0.161 (-0.42, 0.91)

US Oak Ridge workers	Richardson and Wing [58]	n.a. (0 – >0.1)	14,095 (425,486)	IHD (ICD8 410-414)	-2.86 (-6.90, 1.18)
IARC 15- country nuclear worker study	Vrijheid *et al.* [59]	0.0207 (0.0 - >0.5)	275,312 (4,067,861)	Circulatory disease (ICD10 I00-I99, J60-J69, O88.2, R00-R02, R57)	0.09 (-0.43, 0.70)
				IHD (ICD10 I20-I25)	-0.01 (-0.59, 0.69)
				Heart failure (ICD10 I50)	-0.03 (<0, 4.91)
				Deep vein thrombosis and pulmonary embolism (ICD10 I26, I80, I82, O88.2)	-0.95 (-1.00, 9.09)[l]
				CeVD (ICD10 I60-I69)	0.88 (-0.67, 3.16)
				All other circulatory disease (ICD10 R00-R02, R57, I00-I99 excluding I20-26, I50, I60-69, I80, I82)	0.29 (<0, 2.40)
**Environmental studies**					
Three Mile Island study	Talbott *et al.* [60]	0.0001 (0- >0.00016)	32,135 (561,063)	Heart disease (white males)	-274 (-874, 438)
				Heart disease (white females)	-951 (-1433, -390)
Techa River study	Krestinina *et al.* [61]	0.035 (0-0.51)[m]	29,735 (901,563)	All circulatory disease mortality (ICD9 390-459)	0.18 (-0.13, 0.52)[m, h]
				All circulatory disease mortality (ICD9 390-459)	0.24 (-0.08, 0.59)[m, c]
				All circulatory disease mortality (ICD9 390-459)	0.36 (0.02, 0.75)[m, i]
				IHD mortality (ICD9 410-414)	0.26 (-0.22, 0.81)[m, h]
				IHD mortality (ICD9 410-414)	0.40 (-0.11, 0.99)[m, c]
				IHD mortality (ICD9 410-414)	0.56 (0.01, 1.19) [m, i]
Semipalatinsk nuclear test study	Grosche *et al.* [62]	0.09 (0-0.63)[j]	19,545 (582,656)	Heart disease (ICD9 410-429): all settlements	3.22 (2.33, 4.10)[c]
				Heart disease (ICD9 410-429): exposed settlements	0.06 (-0.39, 0.52)[c]
				Stroke (ICD9 430-438): all settlements	2.96 (1.77, 4.14)[c]
				Stroke (ICD9 430-438): exposed settlements	-0.06 (-0.65, 0.54)[c]
				Cardiovascular disease (ICD9 390-459): all settlements	3.15 (2.48, 3.81)[c]
				Cardiovascular disease (ICD9 390-459): exposed settlements	0.02 (-0.32, 0.37)[c]

[a]analysis based on lung dose
[b]analysis based on thyroid dose
[c]assuming a lag period of 10 years.
[d]analysis based on colon dose.
[e]analysis using underlying or contributing cause of death.
[f]analysis based on stomach dose, derived from Table 4 of Yamada *et al.*[21] with smoking and drinking in the stratification.
[g] Risk estimates in relation to cumulative whole body external gamma dose; doses given here are from Moseeva *et al.* [50].
[h] Assuming a lag period of 5 years.
[i] Assuming a lag period of 15 years.
jrisk estimates in relation to cumulative whole body external gamma dose.
[k]90% CI.
[l]estimate derived from log-linear model, evaluated at 1 Sv.
[m]analysis based on dose to muscle.

Supplementary Figure S1. Kaplan-Meier plots by lung dose category and endpoint (<0.05, 0.05-0.19, 0.20-0.29, 0.30-0.49 Gy).

All circulatory disease

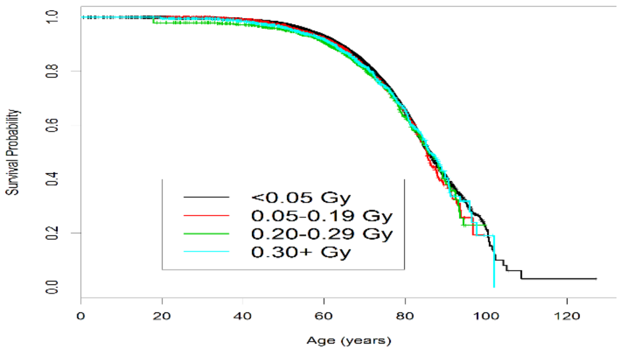

Ischaemic heart disease

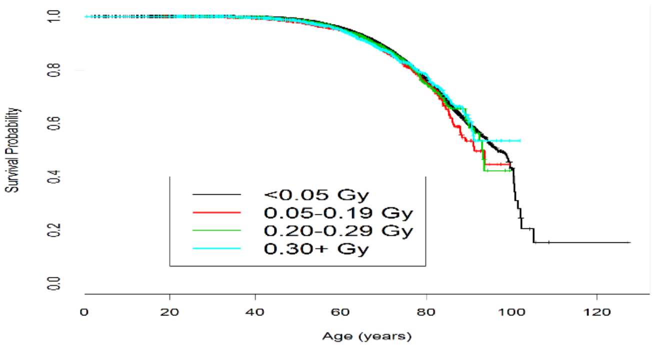

Cerebrovascular disease

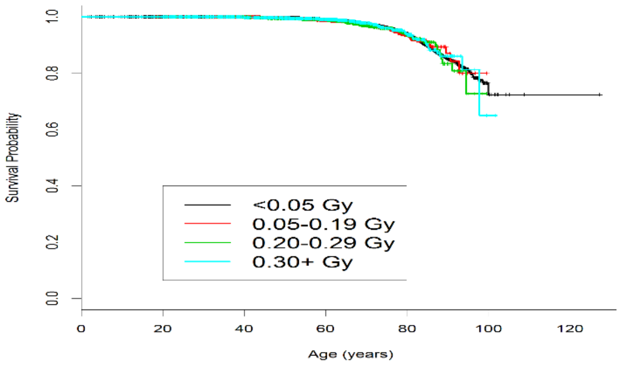

Hypertensive disease

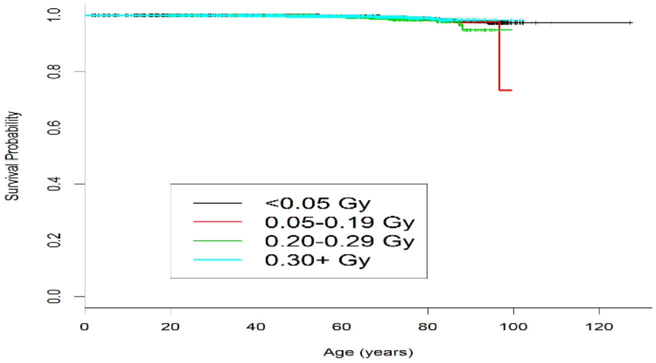

Heart disease other than ischaemic heart disease and hypertensive disease

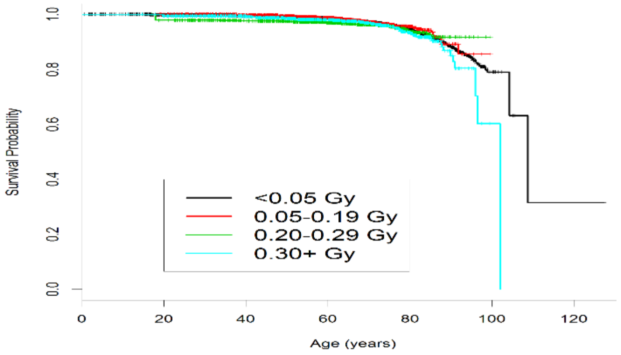

Circulatory disease apart from heart and cerebrovascular disease

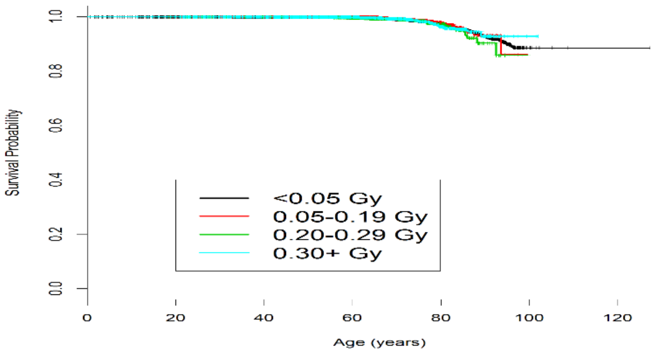

Supplementary Figure S2. Relative risk estimates (and their 95% confidence intervals) against cumulative lagged dose (lagged by 5 years) for the unrestricted dose range. Relative risk estimates are given for cumulative lagged dose categories (0-0.049, 0.050-0.099, 0.100-0.149, 0.150-0.199, 0.200-0.299, 0.300-0.499, 0.500-0.749, 0.750-0.999, 1.000-1.499, 1.500-1.999, 2.000-2.499, 2.500-2.999, 3.000-3.999, 4.000-4.999, 5.000-5.999, ≥6.000 Gy). The dashed red line corresponds to relative risk = 1.

All circulatory disease

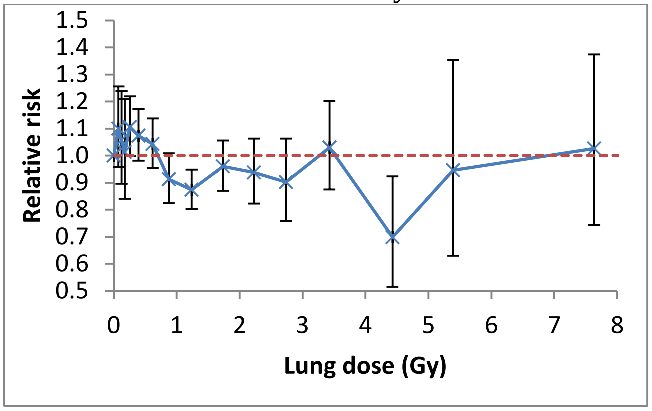

Ischaemic heart disease

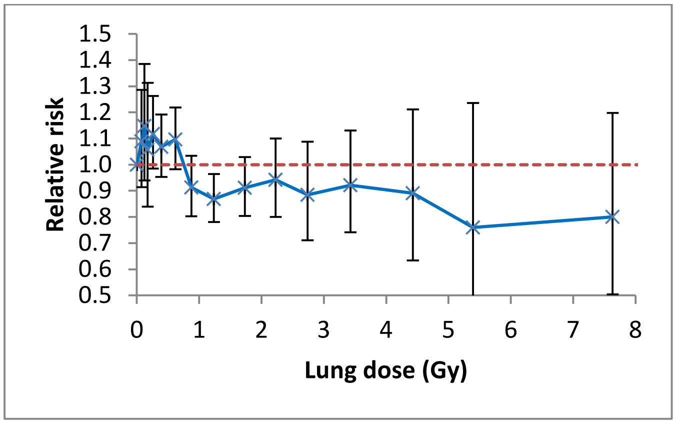

Cerebrovascular disease

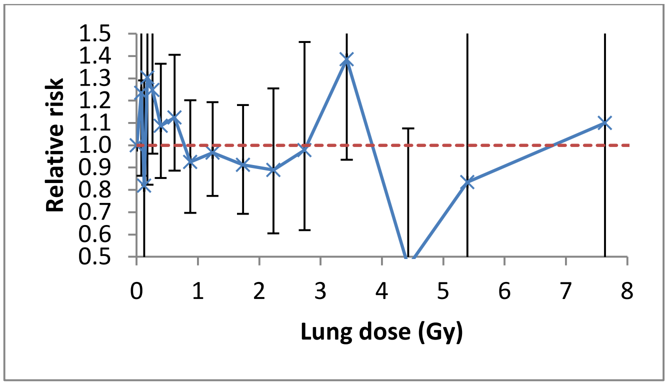

Hypertensive disease

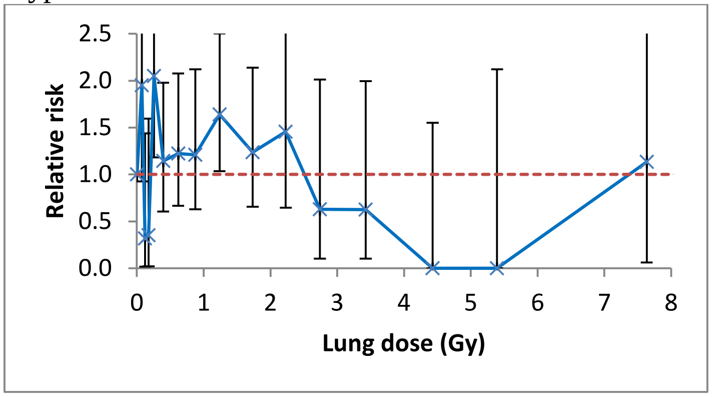

Heart disease other than ischaemic heart disease and hypertensive disease

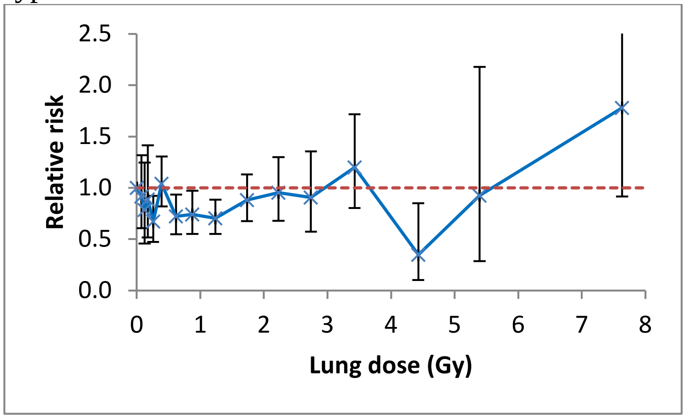

Circulatory disease apart from heart and cerebrovascular disease

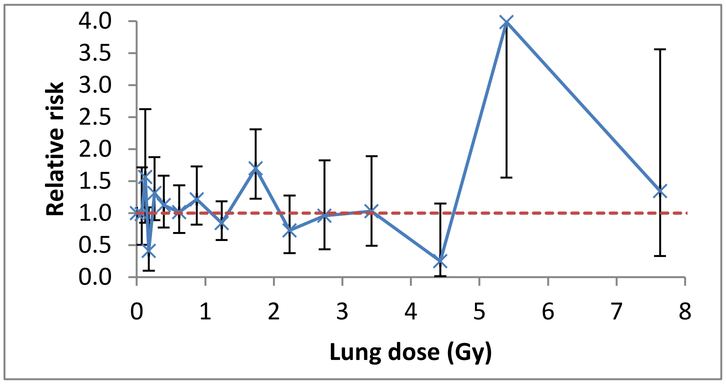

Supplement: Supplementary Tables and Figures [file srep44147-s1.pdf]
